# Integrating multi-omics, EWAS, and reverse network toxicology to explore environmental pollutant risks in erectile dysfunction

**DOI:** 10.3389/fcell.2026.1802191

**Published:** 2026-04-02

**Authors:** Qingtao Yang, Qi Yu, Wei Li, Xi Wei, Jiang Shi, Jun Qiao, Changshi Gu, Fa Sun, Tao Li

**Affiliations:** 1 Department of Urology, the Affiliated Hospital of Guizhou Medical University, Guiyang, China; 2 Department of Reproductive Center, Affiliated Hospital of Guizhou Medical University, Guiyang, China

**Keywords:** environmental pollutants, erectile dysfunction, molecular docking, reverse network toxicology, summary data-based Mendelian randomization

## Abstract

**Background:**

Erectile dysfunction (ED) is increasingly prevalent worldwide, arising from complex interactions between genetic susceptibility and environmental exposure. Real-world exposure involves complex chemical mixtures that may induce synergistic toxicity, which traditional methods struggle to elucidate. This study integrates multi-omics data with reverse network toxicology to systematically identify causal molecular targets and environmental pollutants underlying ED risk, thereby clarifying their mechanisms.

**Methods:**

We performed summary data-based Mendelian randomization (SMR) integrating proteomic (pQTL), transcriptomic (eQTL), and DNA methylation (mQTL) data to identify plasma proteins, gene expression levels, and methylation sites causally linked to ED, with false positives excluded *via* HEIDI tests. The identified targets were used to screen environmental pollutants in the Comparative Toxicogenomics Database Toxicity was predicted using ADMETlab 3.0 and ProTox-III, followed by molecular docking to validate interactions. Functional assays in HUVECs assessed the role of FIS1 and the effects of benzo[a]pyrene.

**Results:**

pQTL-SMR analysis identified 28 plasma proteins significantly associated with ED risk, with consistent effects in both discovery and validation cohorts. Integrated eQTL and mQTL analyses further prioritized FIS1, TNFSF12, and CNP as core targets linked to ED at the protein, gene expression, and methylation. Multi-omics evidence revealed that distinct methylation sites within these genes differentially regulate transcription and translation, exerting different impacts on ED. Using these targets, we screened four environmental pollutants—bisphenol F, tetrabromobisphenol A, benzo[a]pyrene, and chlorpyrifos—as potential regulators. Toxicity predictions indicated mutagenic, cytotoxic, or endocrine-disrupting potential for these compounds. Molecular docking confirmed stable binding to the target proteins (binding free energy ΔG < −5.0 kcal/mol). *In vitro* experiments showed that inhibition of *FIS1* expression suppressed HUVEC proliferation and mitochondrial function, and exposure to benzo[a]pyrene similarly impaired these processes and reduced *FIS1* expression.

**Conclusion:**

This study delineates a potential “environmental pollutant–molecular target–ED” mechanistic pathway, offering new insights into the environmental etiology of ED and establishing a theoretical basis for risk assessment and targeted prevention strategies.

## Introduction

The global prevalence of erectile dysfunction (ED) has risen steadily. Clinically, ED is defined as the persistent inability to achieve or maintain an erection sufficient for satisfactory sexual performance for at least 3 months (Salonia et al., 2021). An estimated 322 million men worldwide are currently affected (Liu et al., 2024). This condition substantially impairs quality of life for patients and their partners and imposes growing burdens on families and healthcare systems (Salonia et al., 2021; Liu et al., 2024; Pantazis et al., 2024). However, the underlying mechanisms of ED remain incompletely understood, and effective preventive strategies are lacking.

ED arises from complex interactions between genetic susceptibility and environmental exposures (Roychoudhury et al., 2021; Zhao et al., 2019). Environmental factors, particularly during critical developmental windows, may exacerbate genetic vulnerabilities and contribute to disease onset (Roychoudhury et al., 2021; Zhao et al., 2019). However, most studies have focused on individual environmental agents. In reality, exposure involves complex chemical mixtures that may produce additive or synergistic toxic effects, challenging traditional risk assessment approaches (Cripps et al., 2021). Therefore, systematically identifying key environmental mixtures and elucidating their molecular targets is essential for advancing understanding of ED pathogenesis and informing effective prevention and intervention strategies.

To address these gaps, integrated computational approaches are increasingly being employed (Dai et al., 2026; Zhao et al., 2025; Zhu T. et al., 2025). Summary data-based Mendelian randomization (SMR) integrates molecular quantitative trait loci to identify methylation sites, genes, and proteins associated with disease susceptibility (Zhu et al., 2016), thereby systematically pinpointing potential causal genes (Jones, 2012; Seem et al., 2024; Kumar et al., 2018). This is complemented by reverse network toxicology, which predict environmental agents targeting these genes or pathways to preliminarily identify pollutants that may influence disease development through epigenetic modifications for subsequent analysis (Qi et al., 2025; Klibaner-Schiff et al., 2024; Kaur et al., 2020; Han and Huang, 2021). Together, this integrated gene–environment framework provides a powerful tool for investigating the interplay between environmental exposures and genetic susceptibility in ED.

Here, we employ an integrated computational strategy to elucidate these mechanisms, providing a theoretical foundation for developing targeted and personalized therapeutic strategies. First, we perform SMR analysis using mQTL, eQTL, and pQTL data—representing DNA methylation, gene expression, and protein levels in blood—to identify targets consistently associated with ED susceptibility across all three omics layers. Subsequently, reverse network toxicology and molecular docking are applied to identify potential environmental pollutants contributing to ED. Finally, additional *in vitro* experiments are conducted to validate the results of the integrated computational strategy.

## Methods and materials

### Summary statistics for eQTL, mQTL, and pQTL

Quantitative trait locus (QTL) data were obtained from the following high-quality cohorts. Gene expression QTL (eQTL) data were sourced from the eQTLGen consortium, which integrates 37 studies comprising 31,684 samples and covers 10,317 SNPs associated with gene expression (Võsa et al., 2021). DNA methylation QTL (mQTL) data were derived from McRae et al., based on two European cohorts totaling 1,980 samples (McRae et al., 2018). Plasma protein QTL (pQTL) data were compiled from two large-scale proteomic projects: the UK Biobank Pharma Proteomics Project (54,219 participants, 2,923 proteins) and the Icelandic cohort (35,559 participants, 4,907 proteins) (Ferkingstad et al., 2021; Sun et al., 2023). The Icelandic cohort served as the discovery set, while the UK Biobank data were used as the replication set. Of note, in subsequent analyses, only complete mQTL and eQTL data corresponding to proteins with consistent associations with ED susceptibility across both discovery and replication cohorts were included.

### ED outcome dataset

Summary statistics for ED were obtained from the genome-wide association study (GWAS) by Bovijn et al. (Bovijn et al., 2019), comprising 223,805 individuals of European ancestry, including 6,175 ED cases. Diagnosis was based on ICD-10 codes (N48.4, F52.2), history of oral ED medication, surgical intervention, or self-report.

### Summary-data-based Mendelian randomization

We performed SMR to identify target associated with ED susceptibility. Using top cis-acting QTLs as instrumental variables, SMR offers greater statistical power than conventional MR when exposure and outcome summary data are derived from large, independent samples (Zhu et al., 2016). The HEIDI test was applied to distinguish pleiotropy from linkage; associations with p < 0.05 were considered driven by linkage disequilibrium and excluded from further analysis (Zhu et al., 2016). Linkage disequilibrium reference data were obtained from the 1000 Genomes Project. All analyses were conducted using SMR software (v1.3.1, Yang Lab, https://yanglab.westlake.edu.cn).

### Environmental pollutant prediction

To identify environmental pollutants potentially associated with ED, we queried the Comparative Toxicogenomics Database (CTD) using the identified causal genes. To ensure biological relevance, we retained only compounds exerting a clear, directional regulatory effect (activation or inhibition) on a given gene. Compounds with conflicting or bidirectional effects, as well as those influencing gene expression only indirectly, were excluded.

### Toxicological property prediction

The toxicological profiles of candidate compounds were systematically evaluated using two computational platforms. ADMETlab 3.0 was used to predict absorption, distribution, metabolism, and excretion (ADME) properties (Fu et al., 2024), while ProTox-III was employed for multi-endpoint toxicity assessment (Banerjee et al., 2024). Screening focused on mutagenicity, cytotoxicity, and endocrine disruption activity. Compounds predicted positive for at least one of these endpoints were retained for further analysis.

### Molecular docking

To further investigate potential interactions between prioritized environmental pollutants and their target proteins, we performed molecular docking simulations. Two-dimensional structures of the compounds were obtained from the PubChem database, and three-dimensional structures of the target proteins were retrieved from the AlphaFold Protein Structure Database. Ligand and receptor structures were prepared using AutoDockTools 1.5.7. Docking simulations were conducted to predict binding poses, calculate binding free energy (ΔG), and assess potential functional impact. A ΔG < 0 kcal/mol indicates a spontaneous binding process, while ΔG < −5.0 kcal/mol suggests a stable interaction.

### Cell culture and treatment

Human umbilical vein endothelial cells (HUVECs) were cultured according to the manufacturer’s protocol in endothelial cell growth medium supplemented with fetal bovine serum, endothelial cell growth supplement, and 1% penicillin/streptomycin, at 37 °C in a humidified atmosphere containing 5% CO_2_. To investigate the role of *FIS1*, gene expression was knocked down using RNA interference. HUVECs were transfected with *FIS1*-targeting siRNA using Lipofectamine 8000 (Beyotime Biotechnology, China) and Opti-MEM (Gibco, United States). The siRNA targeting *FIS1* (si-*FIS1*) was synthesized by Sangon Biotech (Shanghai, China); sequences are provided in the Supplementary Material. To validate the reverse network toxicology predictions, HUVECs were treated with benzo[a]pyrene at a dose consistent with previous literature (Sroczyńska et al., 2022).

### Western blot analysis

Total cellular protein was extracted using RIPA lysis buffer (Solarbio, China) supplemented with protease inhibitors (Yeasen, China). Protein concentration was determined using the Pierce™ BCA Protein Assay Kit (Thermo Fisher Scientific, United States). Equal amounts of protein were separated by electrophoresis on 8%–12% gradient SDS-PAGE gels and transferred to PVDF membranes using a semi-dry transfer system (Bio-Rad, United States). Membranes were blocked with 5% non-fat milk in TBST for 1 h at room temperature, then incubated overnight at 4 °C with primary antibody (anti-FIS1, 1:7000, #10956-1-AP, Proteintech). After washing with TBST, membranes were incubated with horseradish peroxidase-conjugated secondary antibody for 2 h at room temperature. Protein bands were visualized and quantified using Image Lab™ software.

### RNA isolation and quantitative real-time PCR (qRT-PCR)

Gene expression was validated by quantitative real-time PCR (qPCR). Total RNA was extracted using TRIzol reagent, and purity was assessed with a NanoDrop 2000 spectrophotometer (A260/A280 > 1.8). Qualified RNA was reverse-transcribed using the PrimeScript™ RT reagent kit. qPCR was performed on a QuantStudio 5 system with Premix Ex Taq™ reagent kit under the following conditions: initial denaturation at 95 °C for 30 s, followed by 40 cycles of 95 °C for 5 s and 60 °C for 34 s. All reactions were run in triplicate. The primer sequences for *FIS1* are provided in the Supplementary Material.

### EdU proliferation assay

Cell proliferation was assessed using the Cell-Light™ EdU Apollo® 488 in v*itro* kit (Beyotime, China). HUVECs were seeded in 24-well plates at 3 × 10^4^ cells per well and cultured to 70%–80% confluence. Cells were then incubated with 20 μM EdU working solution (Servicebio, China) for 2 h at 37 °C under 5% CO_2_, fixed with 4% paraformaldehyde for 15 min, and permeabilized with 0.5% Triton X-100 for 20 min. EdU-labeled proliferating cells were visualized using the Click-iT™ EdU Alexa Fluor™ 594 imaging kit (Thermo Fisher Scientific, United States) according to the manufacturer’s instructions. Fluorescent images were captured using a Nikon Eclipse Ts2R FL inverted fluorescence microscope with a ×20 objective.

### Statistical analysis

All statistical analyses were performed using R software (version 4.2.2). Differences in gene expression were assessed using the non-parametric Wilcoxon signed-rank test or parametric paired Student’s t-test, as appropriate. A two-sided p-value <0.05 was considered statistically significant. All *in vitro* experiments were performed in at least three independent biological replicates.

## Results

### Proteomic screening for causal proteins in erectile dysfunction

**FIGURE 1:**
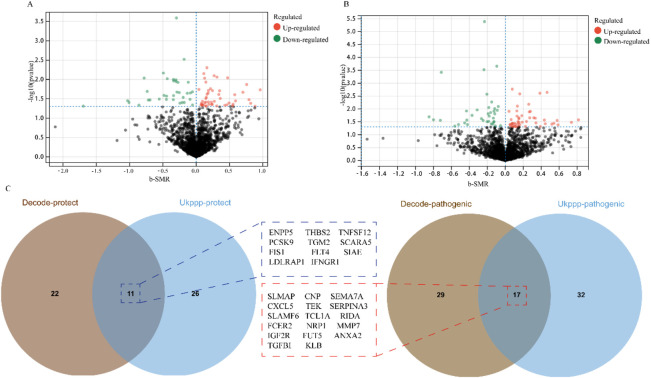
Causal plasma proteins associated with erectile dysfunction susceptibility identified by SMR analysis. **(A)** Volcano plot showing 83 plasma proteins significantly associated with ED susceptibility in the discovery cohort (Icelandic pQTL data). Blue points indicate proteins inversely associated with ED risk (n = 34); red points indicate proteins positively associated with ED risk (n = 49). **(B and C)** Replication of 28 proteins in the UK Biobank Proteomics Project cohort, with 11 showing inverse associations and 17 showing positive associations with ED susceptibility. Full results are provided in Supplementary Tables S1 and S2.

Through SMR analysis (p < 0.05) and HEIDI test screening (p > 0.05), we identified 83 plasma proteins significantly associated with ED susceptibility in the discovery cohort (full results in Supplementary Table S1). Among these, 34 proteins were inversely associated with ED susceptibility, and 49 were positively associated (Figure 1A). In the replication cohort (UK Biobank Proteomics Project), 28 of the 83 proteins were validated: 11 showed an inverse association and 17 showed a positive association with ED susceptibility (Figures 1B,C; complete results in Supplementary Table S2). These findings suggest that these proteins may be functionally interconnected and collectively involved in the pathological processes underlying ED.

### Causal relationship between blood gene expression levels of prioritized proteins and ED

Through SMR analysis (p < 0.05) and further validation by the HEIDI test (p > 0.05), the blood expression levels of four genes, *FIS1*, *TNFSF12*, *CNP*, and *FCER2*, were found to be associated with ED susceptibility. Specifically, the expression of *FIS1* (HR = 0.66, 95% CI: 0.48–0.92, p = 0.013) and *TNFSF12* (HR = 0.85, 95% CI: 0.74–0.97, p = 0.014) was inversely associated with ED susceptibility. Conversely, the expression of *CNP* (HR = 1.18, 95% CI: 1.01–1.39, p = 0.037) and FCER2 (HR = 1.29, 95% CI: 1.02–1.63, p = 0.037) was positively associated with ED susceptibility.

### Causal relationship between blood DNA methylation sites of prioritized proteins and ED

Following SMR analysis (p < 0.05) and validation by the HEIDI test (p > 0.05), 27 methylation sites across nine genes were identified as significantly associated with the genetic susceptibility to ED (Table 1; full results in Supplementary Table S4). These genes include *PCSK9* (8 sites), *KLB* (5 sites), *CXCL5* (2 sites), *FIS1* (1 site), *SCARA5* (4 sites), *ANXA2* (2 sites), *TNFSF12* (1 site), *CNP* (2 sites), and *TGM2* (2 sites). Of these, 18 sites were negatively associated with ED susceptibility, while 9 showed a positive association. Notably, methylation sites cg19802458 in *FIS1* (HR = 0.80, 95% CI: 0.67–0.96, p = 0.016), as well as cg15248157 (HR = 0.96, 95% CI: 0.92–0.99, p = 0.048) and cg16563470 (HR = 0.85, 95% CI: 0.74–0.99, p = 0.035) in *CNP*, were associated with reduced ED susceptibility. In contrast, site cg17892169 in *TNFSF12* (HR = 1.13, 95% CI: 1.03–1.25, p = 0.012) was linked to increased susceptibility. Given that *FIS1*, *CNP*, and *TNFSF12* are all associated with susceptibility to ED at the mQTL, pQTL, and eQTL levels, they were prioritized as key targets influencing ED pathogenesis and selected for subsequent in-depth analysis.

**TABLE 1:** Causal relationship between DNA methylation sites and erectile dysfunction susceptibility.

Probe	Gene	OR	UCI	LCI	p_SMR	p_HEIDI
cg26047355	PCSK9	0.80	0.93	0.68	0.004	0.890
cg14993491	PCSK9	0.81	0.94	0.71	0.004	0.556
cg06197377	PCSK9	0.82	0.94	0.72	0.004	0.851
cg05118916	PCSK9	0.83	0.94	0.73	0.004	0.526
cg26666107	PCSK9	0.81	0.97	0.68	0.020	0.426
cg14977608	PCSK9	1.27	1.51	1.08	0.005	0.616
cg20245116	PCSK9	0.92	0.97	0.88	0.003	0.874
cg13462158	PCSK9	0.93	0.97	0.88	0.003	0.859
cg21880903	KLB	1.28	1.56	1.05	0.017	0.714
cg12523932	KLB	1.08	1.15	1.00	0.045	0.506
cg13732582	KLB	1.08	1.17	1.00	0.045	0.639
cg06235390	KLB	1.08	1.16	1.00	0.045	0.676
cg19786733	KLB	1.07	1.14	1.00	0.045	0.497
cg07868155	CXCL5	0.94	1.00	0.88	0.048	0.475
cg04559909	CXCL5	0.93	1.00	0.87	0.048	0.512
cg19802458	FIS1	0.80	0.96	0.67	0.016	0.857
cg01787494	SCARA5	1.10	1.19	1.02	0.010	0.091
cg09055205	SCARA5	0.86	0.97	0.77	0.011	0.106
cg13709561	SCARA5	0.79	0.97	0.63	0.028	0.341
cg24037389	SCARA5	0.81	0.97	0.68	0.024	0.553
cg13313836	ANXA2	0.88	1.00	0.77	0.049	0.346
cg27554954	ANXA2	0.96	1.00	0.92	0.046	0.683
cg17892169	TNFSF12	1.13	1.25	1.03	0.012	0.253
cg15248157	CNP	0.86	1.00	0.75	0.048	0.376
cg16563470	CNP	0.85	0.99	0.74	0.035	0.449
cg08865458	TGM2	0.96	1.00	0.93	0.029	0.719
cg17352422	TGM2	1.21	1.43	1.02	0.032	0.832

Shown are methylation sites significantly associated with ED, susceptibility following SMR, analysis (p < 0.05) and HEIDI, test validation (p > 0.05). HR, hazard ratio; CI, confidence interval. Full results are provided in Supplementary Table S4.

### The impact of methylation sites of key targets (*FIS1*, *TNFSF12*, and *CNP*) on their gene and protein expression

We next investigated how methylation sites within the core targets, *FIS1*, *TNFSF12*, and *CNP*, influence their gene and protein expression. The results showed that the *FIS1* methylation site cg19802458 was positively correlated with both its gene and protein expression. For *TNFSF12*, the cg17892169 methylation site was negatively associated with its gene and protein expression, although this association did not pass the HEIDI test (p > 0.05). Regarding *CNP*, both methylation sites cg15248157 and cg16563470 were negatively correlated with its gene and protein expression. However, the association between cg15248157 and protein expression did not pass the HEIDI test. These findings suggest that different methylation sites within these genes may influence ED risk through distinct regulatory effects on transcription and translation.

### Initial prediction of environmental compounds

Querying the CTD identified 137, 168, and 98 compounds reported to regulate *CNP*, *FIS1*, and *TNFSF12*, respectively. These included persistent organic pollutants, endocrine disruptors, pesticides, heavy metals, and combustion-related pollutants. Applying our filtering criteria (unidirectional effects consistent with SMR-derived risk associations) retained 30 compounds for *CNP*, 74 for *FIS1*, and 47 for *TNFSF12*. Intersecting these lists revealed four compounds—bisphenol F, tetrabromobisphenol A, benzo[a]pyrene, and chlorpyrifos—that target all three genes. Toxicity predictions indicated that each exhibits mutagenicity, cytotoxicity, or endocrine-disrupting activity. These four were therefore prioritized as potential environmental toxicants that may increase ED risk by modulating CNP, FIS1, and TNFSF12 expression.

### Molecular docking of key targets and environmental compounds

**FIGURE 2:**
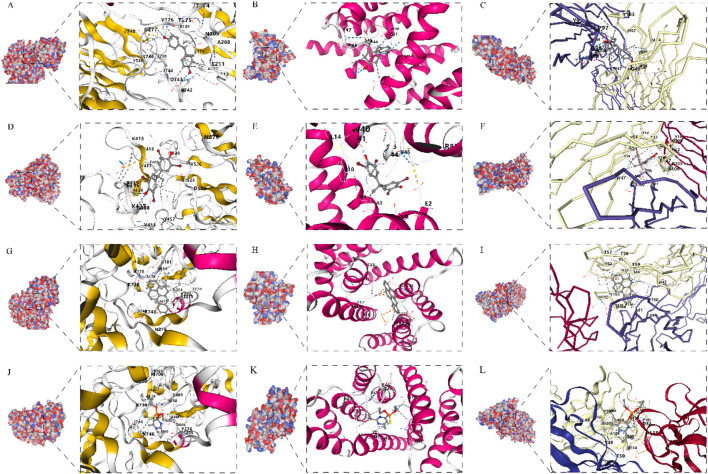
Molecular docking simulations of environmental pollutants with core target proteins. Predicted binding modes and free energies (ΔG) for interactions between the four prioritized pollutants—bisphenol F **(A–C)**, tetrabromobisphenol A **(D–F)**, benzo[a]pyrene **(G–I)**, and chlorpyrifos **(J–L)**—and the three core target proteins: CNP (left column), FIS1 (middle column), and TNFSF12 (right column). All compounds exhibited stable binding with ΔG < −5.0 kcal/mol.

To further validate the reverse toxicology findings, we performed molecular docking to assess binding interactions between the prioritized compounds and the key causal proteins. All four compounds bound stably to the target proteins, with ΔG below −5 kcal/mol (Table 2). Specifically, bisphenol F bound to CNP (−6.7 kcal/mol, Figure 2A), FIS1 (−6.4 kcal/mol, Figure 2B), and TNFSF12 (−7.1 kcal/mol, Figure 2C). Tetrabromobisphenol A bound to CNP (−6.8 kcal/mol, Figure 2D), FIS1 (−5.8 kcal/mol, Figure 2E), and TNFSF12 (−6.7 kcal/mol, Figure 2F). Benzo[a]pyrene bound to CNP (−8.5 kcal/mol, Figure 2G), FIS1 (−7.4 kcal/mol, Figure 2H), and TNFSF12 (−9.6 kcal/mol, Figure 2I). Chlorpyrifos bound to CNP (−6.1 kcal/mol, Figure 2J), FIS1 (−5.6 kcal/mol, Figure 2K), and TNFSF12 (−5.9 kcal/mol, Figure 2L). These results further support that these compounds may contribute to ED pathogenesis through interactions with the three causal targets.

**TABLE 2:** Molecular docking binding free energies between prioritized environmental pollutants and core target proteins.

Compound	Gene	Docking ability
Bisphenol F	CNP	−6.7 kcal/mol
Bisphenol F	FIS1	−6.4 kcal/mol
Bisphenol F	TNFSF12	−7.1 kcal/mol
Tetrabromobisphenol A	CNP	−6.8 kcal/mol
Tetrabromobisphenol A	FIS1	−5.8 kcal/mol
Tetrabromobisphenol A	TNFSF12	−6.7 kcal/mol
Benzo(a)pyrene	CNP	−8.5 kcal/mol
Benzo(a)pyrene	FIS1	−7.4 kcal/mol
Benzo(a)pyrene	TNFSF12	−9.6 kcal/mol
Chlorpyrifos	CNP	−6.1 kcal/mol
Chlorpyrifos	FIS1	−5.6 kcal/mol
Chlorpyrifos	TNFSF12	−5.9 kcal/mol

Binding free energy (ΔG) was calculated using AutoDockTools 1.5.7. ΔG < −5.0 kcal/mol indicates stable binding. All four compounds—bisphenol F, tetrabromobisphenol A, benzo[a]pyrene, and chlorpyrifos—formed stable interactions with CNP, FIS1, and TNFSF12.

### *In vitro* validation of the core protein FIS1

**FIGURE 3:**
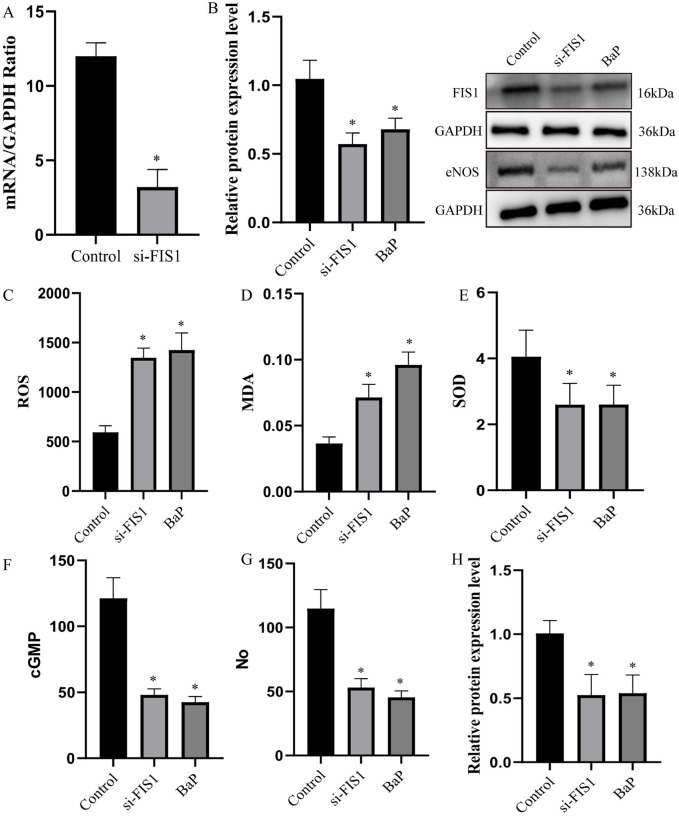
Functional validation of FIS1 and effects of benzo[a]pyrene in HUVECs. **(A and B)** qPCR and western blot confirming effective knockdown of *FIS1* expression at mRNA and protein levels. **(C–E)** Effects of *FIS1* knockdown and benzo[a]pyrene (BaP) exposure on oxidative stress markers: reactive oxygen species (ROS) levels **(C)**, malondialdehyde (MDA) levels **(D)**, and superoxide dismutase (SOD) activity **(E)**. **(F–H)** Effects on endothelial function markers: cyclic guanosine monophosphate (cGMP) levels **(F)**, nitric oxide (NO) levels **(G)**, and endothelial nitric oxide synthase (eNOS) protein expression **(H)**. Data are presented as mean ± SEM from three independent experiments. *p < 0.05, **p < 0.01, ***p < 0.001 compared to control group.

**FIGURE 4:**
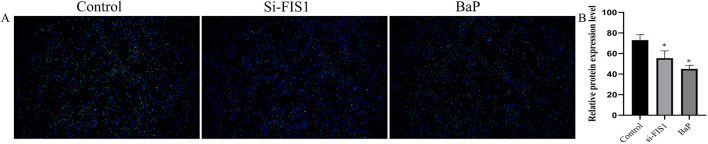
Effects of FIS1 knockdown and benzo[a]pyrene exposure on HUVEC proliferation. **(A)** Representative fluorescence images of EdU incorporation assay showing proliferating HUVECs (red) and total nuclei (blue) under control, si-*FIS1*, and benzo[a]pyrene (BaP)-treated conditions. Scale bar, 100 μm. **(B)** Quantification of EdU-positive cells, expressed as percentage of total cells. Data are presented as mean ± SEM from three independent experiments. **p* <0.01 compared to control group.

To validate the SMR findings, we performed functional experiments in HUVECs. qPCR and western blot confirmed effective knockdown of *FIS1* at both mRNA and protein levels (Figures 3A,B). Functionally, FIS1 knockdown increased levels of pro-oxidant factors (ROS and MDA) and decreased SOD activity (Figures 3C–E), indicating oxidative stress—a hallmark of mitochondrial dysfunction. Moreover, ELISA and western blot revealed that *FIS1* downregulation reduced cGMP and NO levels, as well as eNOS protein expression (Figures 3F–H). EdU assays further showed that *FIS1* knockdown significantly suppressed endothelial cell proliferation (Figure 4). Together, these results support a protective role for FIS1 in endothelial function and ED pathogenesis.

To further validate the reverse network toxicology and docking predictions, we selected benzo[a]pyrene—the pollutant with the most stable docking scores—for intervention experiments. Benzo[a]pyrene exposure significantly suppressed *FIS1* expression and impaired endothelial cell function (Figures 3C–H,4), experimentally confirming that this pollutant may disrupt endothelial homeostasis by targeting FIS1. These findings support the proposed “environmental pollutant–FIS1–mitochondrial function–ED” pathway.

## Discussion

ED is a growing global health concern, with a steadily rising prevalence that imposes substantial burdens on society and healthcare systems (Salonia et al., 2021; Liu et al., 2024; Pantazis et al., 2024). Its pathogenesis is complex, arising from multifactorial interactions between genetic susceptibility and environmental exposures (Roychoudhury et al., 2021; Zhao et al., 2019; Cripps et al., 2021). Environmental factors, particularly during critical developmental windows, can amplify genetic risks and trigger disease onset (Roychoudhury et al., 2021; Zhao et al., 2019; Cripps et al., 2021). Real-world exposures, however, involve complex chemical mixtures that may exert additive or synergistic toxic effects, complicating the elucidation of pathogenic mechanisms (Zhao et al., 2019; Cripps et al., 2021). Therefore, systematically identifying key environmental mixtures and their molecular targets is essential for advancing our understanding of ED pathology and for informing effective prevention and intervention strategies.

To this end, we first performed pQTL-based SMR analysis and identified 28 plasma proteins significantly associated with ED risk. Subsequent integration of eQTL and mQTL data pinpointed three core targets—*FIS1*, *TNFSF12*, and *CNP*—that were significantly associated with ED at the protein, gene expression, and DNA methylation levels. For FIS1, methylation at cg19802458, together with its gene expression and protein abundance, was consistently associated with reduced ED susceptibility, providing coherent multi-omics evidence. Integrated analysis further elucidated regulatory relationships among these targets: methylation at the *FIS1* cg19802458 site was associated with increased gene and protein expression. In contrast, distinct methylation sites in *CNP* and *TNFSF12* exhibited divergent regulatory patterns, suggesting a complex epigenetic network modulating ED susceptibility. In summary, leveraging population-scale multi-omics data, this study is the first to reveal causal associations of *FIS1*, *CNP*, and *TNFSF12* with ED susceptibility and to preliminarily elucidate their epigenetic regulatory pathways, offering new insights into the molecular mechanisms underlying ED.

*FIS1* encodes mitochondrial fission 1 protein, a key regulator of mitochondrial fission that influences mitochondrial quantity, morphology, cellular function, and survival (Cerveny et al., 2007). Dysregulation of *FIS1* has been implicated in cancer, metabolic diseases, and neurodegenerative disorders (Qin et al., 2025; Waters et al., 2022; Rovira-Llopis et al., 2017), but its role in ED remains underexplored. A central mechanism in ED is vascular endothelial dysfunction, which reduces nitric oxide synthesis, impairs hemodynamics, and compromises erectile function (MacDon et al., 2021; Zhu D. et al., 2025; Zhuang et al., 2025). Emerging evidence suggests that mitochondrial dysfunction contributes to ED, with oxidative stress-induced mitochondrial damage and apoptosis potentially undermining vascular repair and exacerbating disease progression (Gómez Del Val et al., 2025; Yang et al., 2025; Yang et al., 2022). Notably, recent findings indicate that upregulation of mitochondrial FIS1 ameliorates aging-related endothelial dysfunction in endothelial progenitor cells by enhancing ATP production and reducing oxidative stress, thereby improving cell viability and vascular function (Wang et al., 2019). Consistent with these observations, our findings suggest that higher *FIS1* expression may lower ED risk by preserving mitochondrial homeostasis, supporting endothelial function, and maintaining normal hemodynamics.

Similar to *FIS1*, *CNP* also plays a key role in regulating mitochondrial fission (Olga et al., 2020; Tan et al., 2021). As an enzyme, CNP catalyzes 2′,3′-cyclic nucleotides, which have been implicated in impairing mitochondrial integrity and promoting opening of the mitochondrial permeability transition pore (mPTP) (Olga et al., 2020; Tan et al., 2021). As a mitochondria-associated protein, *CNP* may also influence mitochondrial respiration and energy production through interactions with respiratory chain complexes I–V in the inner mitochondrial membrane (Olga et al., 2020; Tan et al., 2021; Azarashvili et al., 2009). Direct evidence linking *CNP* to ED remains limited; its potential detrimental effects may involve disruption of mitochondrial homeostasis in endothelial cells, warranting further investigation.

*TNFSF12* encodes the TNF-like weak inducer of apoptosis (TWEAK), which binds its receptor TNFRSF12A to regulate diverse biological processes (Ratajczak et al., 2022). As with the two targets above, direct evidence for its involvement in ED is not well established. However, vascular endothelial dysfunction is a central mechanism in ED, leading to reduced nitric oxide synthesis and impaired hemodynamics (Zhuang et al., 2025; Gómez Del Val et al., 2025; Yang et al., 2025; Yang et al., 2022). TWEAK has been shown to activate cellular responses including proliferation, migration, and angiogenesis, and is implicated in maintaining normal endothelial cell function (Ratajczak et al., 2022; Tong et al., 2024; Wajant, 2013)^.^ This suggests that *TNFSF12* may exert a protective effect against ED by supporting endothelial cell function, proliferation, and survival.

Building on the causal associations of *FIS1*, *CNP*, and *TNFSF12* with ED, this study identified four environmental pollutants—bisphenol F, tetrabromobisphenol A, benzo[a]pyrene, and chlorpyrifos—from the CTD database as predicted regulators of these three key genes. Previous studies have implicated these compounds in ED through oxidative stress, endocrine disruption, and apoptosis (Roychoudhury et al., 2021; Zhao et al., 2019; Cripps et al., 2021), but their precise molecular mechanisms remain unclear. Although some of these pollutants exhibit mitochondrial-targeting effects in other disease models (Reddam et al., 2022; Lambertini and Byun, 2016), their specific roles in ED require further investigation. Our findings suggest that these pollutants may contribute to ED pathogenesis by disrupting mitochondrial function, with *FIS1* and *CNP* as key mediators, and by impairing endothelial function, involving *TNFSF12*. Molecular docking confirmed stable binding of all four compounds to the three target proteins (ΔG < −5.0 kcal/mol), supporting potential direct interactions. *In vitro* experiments further demonstrated that these compounds impair endothelial cell function and mitochondrial status, validating the critical regulatory role of FIS1. Collectively, this work provides important insights into the “environmental exposure–molecular target–ED” mechanistic pathway and establishes an experimental and theoretical foundation for developing targeted intervention strategies.

It is also noteworthy that our multi-omics analysis revealed a positive association between methylation at cg19802458 and *FIS1* expression. According to K450 annotation, this CpG site is located in an intergenic region approximately 388 bp downstream of the *FIS1* gene body (GRCh37/hg19: chr7:100882739–100895597). The classical model of gene-body methylation posits that intragenic methylation can promote transcription by suppressing cryptic promoters or facilitating elongation (Jones, 2012; Seem et al., 2024; Kumar et al., 2018); however, this mechanism typically applies to sites within the transcribed region. Since cg19802458 lies outside the gene body, its regulatory effect is unlikely to be explained by canonical gene-body methylation. Emerging evidence suggests that intergenic methylation may influence gene expression through alternative mechanisms, such as modulating distal enhancer activity, altering CTCF-mediated chromatin looping, or regulating transcription of overlapping or nearby non-coding RNAs (Ehrlich and Lacey, 2013; Meng et al., 2015; Moore et al., 2013). Thus, the observed correlation between cg19802458 methylation and increased *FIS1* expression may reflect a more complex, distal regulatory architecture rather than direct intragenic epigenetic control.

The primary strength of this study lies in its integrative multi-method strategy, which strengthens causal inference linking key genes to ED risk. The SMR approach effectively reduces confounding and mitigates reverse causation. The subsequent integration of reverse network toxicology, molecular docking, and *in vitro* experiments not only provides mechanistic insights into how mixed environmental pollutants may influence ED through specific targets (*FIS1*, *CNP*, and *TNFSF12*), but also opens new avenues for identifying early biomarkers and developing targeted interventions.

Although this study integrates multi-omics and reverse network toxicology approaches and provides new insights into environmental pollutant contributions to ED, several limitations should be considered when interpreting the results. First, at the population level, the ED-GWAS and eQTL, mQTL, and pQTL data used were predominantly derived from European populations. This reflects the current availability of large-scale genetic resources; sample sizes for non-European populations remain insufficient to support robust cross-ancestry analyses. Future studies should leverage multi-ancestry genetic resources to validate the generalizability of the core targets.

Second, regarding exposure assessment, pollutant screening was based on *in silico* predictions from the CTD database. While this approach effectively identifies candidate compounds, it cannot account for the combined effects of exposure mixtures or inter-individual variability in real-world settings. To partly address this limitation, we confirmed stable binding between pollutants and target proteins through molecular docking and selected benzo[a]pyrene for subsequent *in vitro* functional validation. Future research should adopt exposomic approaches by utilizing pollutant concentrations measured in human biosamples to validate associations in more realistic exposure settings.

Third, regarding causal inference methodology, we employed SMR analysis with strict use of *cis*-QTLs as instrumental variables and applied the HEIDI test (p > 0.05) to exclude false positives due to linkage disequilibrium, enhancing reliability. However, this method cannot completely eliminate unmeasured confounding, horizontal pleiotropy, or weak instrument bias. Future studies should further validate the robustness of genetic associations using colocalization analysis, multivariable MR, or prospective cohort studies.

Fourth, regarding experimental validation, we confirmed the binding of four pollutants to three target proteins through molecular docking and prioritized *FIS1* for *in vitro* validation, given its consistent associations with ED risk across all three omics levels and its potential involvement in mitochondrial function. However, we did not conduct equally detailed mechanistic experiments for *CNP*, *TNFSF12*, or the other three pollutants. Furthermore, although SMR suggested associations between *FIS1* methylation sites and ED, the mechanisms by which pollutants influence gene expression through epigenetic or transcriptional regulation remain to be explored. Future research should conduct systematic functional experiments for each target and pollutant, integrating high-throughput techniques such as ChIP–seq and methylation sequencing to construct regulatory networks linking pollutants, target genes, and downstream pathways.

Finally, regarding cellular models, we selected HUVECs based on the central role of endothelial dysfunction in ED pathogenesis. However, ED involves synergistic interactions among multiple cell types, including corpus cavernosum smooth muscle cells and neuronal cells. Future studies should establish primary corpus cavernosum smooth muscle cell models, endothelial-smooth muscle co-culture systems, and incorporate animal models to evaluate target function and its impact on erectile function at the organismal level.

## Conclusion

By integrating multi-omics data with a reverse network toxicology approach, this study systematically delineates the molecular connections between environmental pollutants and ED. We identified *FIS1*, *CNP*, and *TNFSF12* as key targets influencing ED risk, with their expression subject to epigenetic regulation. Furthermore, we pinpointed four environmental pollutants—bisphenol F, tetrabromobisphenol A, benzo[a]pyrene, and chlorpyrifos—that may increase ED risk by disrupting endothelial function and mitochondrial homeostasis through these targets. Molecular docking and *in vitro* experiments confirmed stable binding of these pollutants to the target proteins and their corresponding biological effects. This work provides novel insights into the environmental etiology of ED and establishes a theoretical foundation for developing early prevention and targeted intervention strategies.

## Data Availability

The original contributions presented in the study are included in the article/Supplementary Material, further inquiries can be directed to the corresponding authors.
